# The pipeline starts in medical school: characterizing clinician-educator training programs for U.S. medical students

**DOI:** 10.1080/10872981.2022.2096841

**Published:** 2022-07-07

**Authors:** Ryan C. Bahar, Aidan W. O’Shea, Eric S. Li, Madisen A. Swallow, August A. Allocco, Judy M. Spak, Janet P. Hafler

**Affiliations:** a School of Medicine, Yale University,New Haven, CT, USA; b School of Medicine and Dentistry, University of Rochester, Rochester, NY, USA; c Yale School of Medicine, Cushing/Whitney Medical Library, New Haven, CT, USA; d Yale School of Medicine, Teaching and Learning Center, New Haven, CT, USA

**Keywords:** Clinician-educator, undergraduate medical education, career development, scholarly concentration, medical student

## Abstract

In the past forty years, clinician-educators have become indispensable to academic medicine. Numerous clinician-educator-training programs exist within graduate medical education (GME) as clinician-educator tracks (CETs). However, there is a call for the clinician-educator pipeline to begin earlier. This work aims to identify and characterize clinician-educator track-like programs (CETLs) available in undergraduate medical education (UME). We developed an algorithm of 20 individual keyword queries to search the website of each U.S. allopathic medical school for CETLs. We performed the web search between March to April 2021 and repeated the search between July and September 2021. The search identified CETLs for 79 (51%) of the 155 U.S. allopathic medical schools. The identified CETLs commonly address the clinician-educator competency of educational theory (86%, 68/79), are formally organized as concentrations or analogous structures (52%, 41/79), and span all four years of medical school (37%, 29/79). The prevalence of CETLs varies with geography and medical school ranking. We provide an overview of the current state of CETLs as assessed from institutional websites. To create a future with a sustainable output of skilled clinician-educators, UME must continue to increase the number and quality of CETLs.

## Introduction

In the past forty years, clinician-educators have become indispensable to the medical education enterprise [1,2]. Clinician-educators comprise the majority of academic physicians [3], fulfill both educational and clinical missions of academic medical centers [4], and receive recognition through distinct promotional tracks [1,5]. A recent consensus definition for the 21st-century clinician-educator [6] highlights the multifaceted nature of the role: an individual who participates in clinical practice, applies theory to education practice, engages in scholarship, and consults on education issues. Indeed, with major national organizations such as the Carnegie Foundation calling for significant reforms to medical training [7], a sustainable pipeline of clinician-educators who are education scholars – in addition to being exceptional teachers – is necessary to advance medical education [8,9].

However, the current paradigm of undergraduate medical education (UME) rarely teaches students the educator skills essential for the practice of future clinician-educators [6,10,11]. Clinician-educators often must pursue master’s or doctoral programs in health professions education [12,13], faculty development programs [14], and/or post-residency medical education fellowships [15]. Over the past two decades, clinician-educator-training programs have begun appearing in residency programs as clinician-educator tracks (CETs) [16,17]. This emergence may be due to the recognition that residents skilled in clinician-educator competencies are needed to soon join the clinician-educator workforce. Moreover, enhanced recruitment of future clinician-educators in residency may counteract low faculty clinician-educator retention rates [18], thereby bolstering the number of clinician-educators at academic medical centers [16]. This is in addition to the ongoing need to develop resident teaching skills – up to one third of medical student teaching is performed by residents [19] – coupled with accreditation organization requirements for resident teaching [20,21].

Recent literature has called for the clinician-educator pipeline to begin earlier than residency: in undergraduate medical education [22,23]. Cultivating educator identities in medical students may be a solution for generating sustained interest in the clinician-educator pathway during training. Exposure to and identification with educator role models fosters educator identity and produces future clinician-educators, according to the community of practice framework [24,25]. Furthermore, clear career pathways and early research exposure are known to positively influence the choice to become an academic physician [26,27].

The 2020 supplement to Academic Medicine [28] highlights the recent expansion of student enrichment tracks in UME. Other works document the rise in scholarly concentration programs, including those in medical education [29,30]. These emerging formal structures show promise for extending the clinician-educator pipeline into earlier stages of medical training, encouraging educator identity formation in medical students as CETs do for residents. Our study will refer to these structures for medical students as clinician-educator track-like programs (CETLs).

Existing literature mainly centers on programs that train students to be better teachers [10,31,32]. However, with increasing attention in UME to programs developing the educator skills (e.g., scholarship) and knowledge (e.g., pedagogical theory) vital to the 21st-century clinician-educator [6,33–35], a comprehensive study of the current state of CETLs nationwide is needed. This work aims to identify and characterize CETLs in UME at U.S. allopathic medical schools in order to understand how medical schools prepare students for careers as clinician-educators and to inform future directions for CETL development.

## Materials and methods

### Algorithmic search overview

Four trained medical student researchers (A.A.A., R.C.B., E.S.L., M.A.S.) performed an algorithmic web search of all 155 U.S. allopathic medical schools to identify those with existing CETLs in March-April 2021. Three researchers (R.C.B., E.S.L., M.A.S.) repeated the search in July-September 2021. Prior to the search, we obtained the list of U.S. allopathic medical schools from the Liaison Committee on Medical Education (LCME) website [36]. We independently executed the search algorithm for distinct school lists and exchanged schools for the second iteration of the search. In developing the search algorithm, we adhered to established recommendations for conducting systematic web searches [37]. Prior works studying UME scholarly concentration programs [29,38] and reviewing medical school websites [39] informed our search methodology.

### CETL definition and exclusion criteria

Using the clinician-educator definition of Sherbino and colleagues [6] and criteria for CETs established by Friedman and colleagues [16], we defined CETLs as programs that target UME learners and address three core clinician-educator competencies: direct teaching, educational scholarship, and educational theory. We categorized CETLs as full or partial depending on the number of criteria met. Full CETLs meet all 4 criteria (longitudinal, direct teaching, educational scholarship, educational theory) whereas partial meet 3 or fewer (Table 1). We established criteria to exclude specialty- and knowledge-specific programs, non-dual degree programs in education, and programs meeting specific CETL criteria that are not substantive enough to constitute a CETL (Table 2).

**Table 1.** Definition of a clinician-educator track-like (CETL) program in undergraduate medical education (UME).

CETL Program Definition: a longitudinal (≥4-week) program targeting UME learners that addresses 3 core clinician-educator competencies	
Direct Teaching	Effectively uses scholarly teaching techniques in the clinical and extraclinical environments
Educational Scholarship	Contributes to the development, dissemination, and translation of health professions knowledge and practice
Educational Theory	Maintains knowledge of education theory, psychology, and principles, and applies this knowledge to education practice
*Full CETL programs fulfill all the above criteria; partial CETL programs lack one or more criteria. Dual degree education programs are included in analyses for schools without a full CETL program

**Table 2.** Exclusion criteria for clinician-educator track-like (CETL) programs.

CETL Exclusion Criteria:
Specialty-specific programs Programs requiring skill/knowledge prerequisites not shared by all medical students at the targeted level of training Degree programs in education not listed as dual degree offerings Programs meeting the ‘longitudinal (≥4-week) program’ criterion and/or ‘direct teaching’ criterion without meeting any other criteria

### Web search algorithm

**Figure 1.**
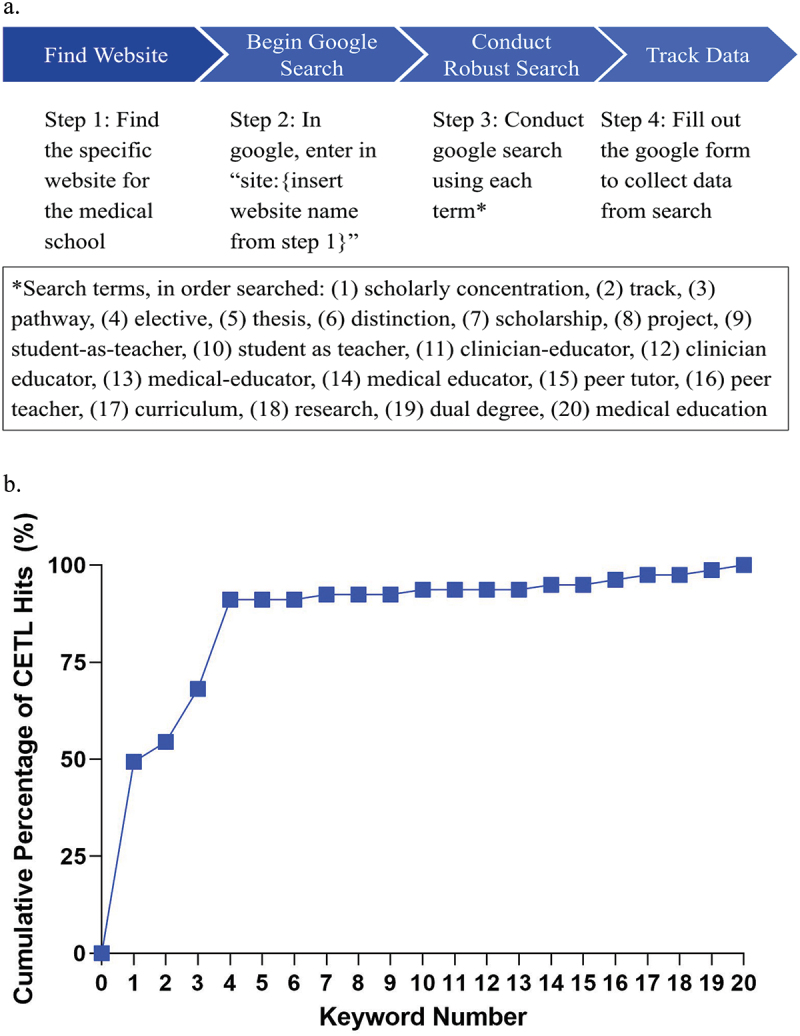
(a) Web search algorithm. (b) Cumulative percentage of clinician-educator track-like (CETL) programs identified after each search term.

The web search used an algorithmic approach (Figure 1). We first located the website for each medical school through the Medical School Admission Requirements (MSAR) online database [40] and confirmed it with a Google search. Entering the website name into the Google search bar (‘site:[website name]’) limited the search to the institutional website. We conducted a series of queries using 20 keywords, in order, until we identified a full CETL for each school. We chose keywords in consultation with an experienced research librarian (J.M.S.). For each keyword query, we clicked on all links on the first page of Google results and scanned the directly linked web pages for content that fit our CETL criteria.

If an individual keyword query was negative (i.e., did not uncover content consistent with a CETL), we continued through the keyword list until a query was positive or until all keywords were exhausted. When a keyword query was positive, we extracted the following information in a Google form: (1) keyword resulting in CETL hit; (2) website link for CETL; (3) number of CETL criteria filled; (4) type of CETL (full/partial); (5) brief program description; (6) length and timing of program; and (7) explanation of how the program fulfills ‘direct teaching,’ ‘educational scholarship,’ and/or ‘educational theory’ criteria. We also recorded when all keyword queries were exhausted for a school without hitting a CETL program (Figure 1A).

### Data analysis

We reviewed extracted data as a group and calculated Cohen’s kappa statistics to measure inter-rater reliability. Group discussion resolved disagreements and ensured consensus. An independent reviewer (A.W.O.) verified and compiled CETL information for each institution post-extraction. We performed descriptive data analysis in Google Sheets and identified medical school subgroups by institution characteristics (geographic region, private/public, ranking). Two-sample z-tests for independent proportions assessed for subgroup differences in CETL availability.

## Results

### Web search

We analyzed inter-rater reliability during the two iterations of the search to evaluate reproducibility. We observed a median percent agreement of 87% for 2 raters performing the web search algorithm for the same institutions (range across 5 rater pairs: 80–100%) and a Cohen’s kappa of 0.75 (range: 0.59–1.00). This reflects substantial agreement between raters in finding CETLs through the web search [41]. More detailed reporting of inter-rater reliability can be found in Table A1 in the Appendix.

We also examined when CETLs were identified during the execution of the search algorithm. Forty-nine percent (39/79) of CETLs were identified after 1 keyword search: ‘scholarly concentration.’ Ninety-one percent (72/79) of CETLs were identified within 4 keyword searches (Figure 1B).

### CETL identification

**Figure 2.**
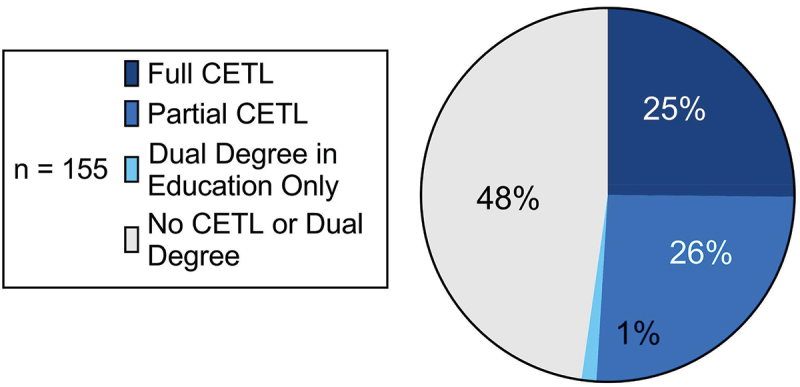
Clinician-educator track-like (CETL) program availability at U.S. allopathic medical schools.

The web search identified CETLs at 79 (51%) of the 155 U.S. allopathic medical schools. Thirty-nine (25%) have full CETLs; 40 (26%) have partial CETLs, including 3 that also offer a dual degree in education; and 2 (1%) do not have CETLs but offer a dual degree in education (Figure 2). Individual program summaries and links for all 79 CETLs identified in this study are in Table 3.

**Table 3.** Identified clinician-educator track-like (CETL) programs by institution.

Institution Name	Institution Type	Program URL	CETL Program Type	CETL Program Name	Program Length	Program Timing	Program Description	CETL Classification	L*	DT*	ES*	ET*
Albert Einstein College of Medicine	Private	https://einsteinregistrar.org/current-students/elective-course-desc/	Elective	Student as Teacher	4 weeks	M4	Four-week M4 elective that ‘consolidate[s]’ fourth year teaching opportunities. Students work with a faculty member on a ‘curricular enhancement project,’ and the elective provides a didactic curriculum on principles of adult learning.	Full	✓	✓	✓	✓
Boston University School of Medicine	Private	http://www.bumc.bu.edu/busm/education/registrars-office/third-and-fourth-year-scheduling/teachingscholarly-activities/	Elective	Medical Education Curriculum Projects	4 weeks	M4	‘[The] elective provides for a MS4 student the opportunity to use a systematic instructional design process to discover, design, develop, and deploy in four weeks an instructional project on a content area of their choice.’	Partial	✓	-	✓	-
Brody School of Medicine at East Carolina University	Public	https://medicine.ecu.edu/medicaleducation/met/	Concentration/Pathway/Project/**Track**	Medical Education & Teaching Distinction Track	All 4 years	M1-M4	Longitudinal program incorporating a medical education lecture series and direct teaching (≥40 hours) and culminating in the production and presentation of ‘enduring educational materials or an educational research manuscript submitted for publication.’	Full	✓	✓	✓	✓
California Northstate University College of Medicine	Private	https://medicine.cnsu.edu/PDFs/M4-Catalog_2019-2020.pdf	Elective	Cardiovascular & Pulmonary TA	4 weeks	M4	Two- or four-week M4 elective offering the opportunity to teach in mini-lectures and review sessions; elective also provides facilitator training for students to understand and apply various teaching pedagogies.	Partial	✓	✓	-	✓
Charles E. Schmidt College of Medicine at Florida Atlantic University	Public	http://med.fau.edu/research/medical-students/opportunities.php	Research Program	Medical Education Research, Scholarship, and Evaluation Research Opportunity	>4 weeks – 1 year	M1 Summer or M2 Summer	‘Fast track,’ summer-long M1/M2 research opportunity; students are assigned to a study on pedagogical and/or sociocultural determinants of medical educational outcomes and submit their research to a regional or national conference.	Partial	✓	-	✓	-
Columbia University Vagelos College of Physicians and Surgeons	Private	https://www.vagelos.columbia.edu/file/32821/download?token=ONEeazoT	Concentration/Pathway/**Project/**Track	Medical Education Scholarly Project	>4 weeks – 1 year	M3-M4	Offering within required Scholarly Projects Program providing at least 4 months of protected time to complete a scholarly medical education project, teach in existing courses and programs, create curricular content, and attend scholarly project didactic sessions.	Full	✓	✓	✓	✓
Creighton University School of Medicine	Private	https://catalog.creighton.edu/medicine/curriculum/#courseinventory	Elective	Medical Education Elective	4 weeks	M4	Two- or four-week M4 elective introducing students to general topics in teaching and medical education scholarship and providing instruction in teaching skills. Provides support for the development of a scholarly project.	Partial	✓	-	✓	✓
David Geffen School of Medicine at UCLA	Public	https://ucla.oasisscheduling.com/public/view_course.html?yid=2022;did=EDU;cid=MD150.02	Elective	Medical Education: Teaching Fellowship	>4 weeks – 1 year	M4	Yearlong M4 ‘thread’ elective for students to co-facilitate small group learning with a faculty member. Students also participate in a series of interactive seminars on learning theory and teaching practices.	Partial	✓	✓	-	✓
Donald and Barbara Zucker School of Medicine at Hofstra/Northwell	Private	https://medicine.hofstra.edu/pdf/education/md/education-ms4-course-catalog.pdf	Elective	Introduction to Medical Education: Medical Student as Teacher	4 weeks	M4	Two-part M4 elective that first engages students in a ‘bootcamp’ on medical education theory and practice, then involves them in 20 hours of direct teaching. Students also participate in journal club, complete a final scholarly presentation, and are exposed to modern tools in medical education.	Full	✓	✓	✓	✓
Drexel University College of Medicine	Private	webcampus.drexelmed.edu/clinicaleducation/SeniorElectives/2021-2022/default.asp?Action=Listing&CourseNo=OMED8632S410&Institution=Drexel&#±&#University&#±&#College&#±&#of&#±&#Medicine	Elective	Projects in Medical Education	2 weeks or less	M4	Two-week M4 elective where students produce a work of medical educational scholarship, from formulating a testable hypothesis to writing up the project in a form suitable for submission to a journal and giving an oral presentation.	Partial	-	-	✓	-
Duke University School of Medicine	Private	https://medschool.duke.edu/education/health-professions-education-programs/doctor-medicine-md-program/curriculum/third-year-18	Research Program	Medical Education Research Program	>4 weeks – 1 year	M3	Yearlong M3 research program where students perform a medical education research project under a mentor, receive statistics training, prepare a thesis, and present their research. A didactic component provides instruction on educational theory and methodology, and students are also involved in direct teaching.	Full	✓	✓	✓	✓
Eastern Virginia Medical School	Public	https://06ff3566-a318-4102-a985-6521bc798e7a.filesusr.com/ugd/572910_12ed3ebe6e7b45b5a246d429b32ea036.pdf	Elective	Student Academic Clinician Educator	4 weeks	M4	Longitudinal elective (unspecified duration but for 4 total weeks of credit) involving didactic workshops on educational theory, direct teaching or mentoring (≥10 hours), and generation of a personalized teaching philosophy statement.	Partial	✓	✓	-	✓
Frank H. Netter MD School of Medicine at Quinnipiac University	Private	https://www.qu.edu/schools/medicine/programs/medical-doctor-degree/md/curriculum/scholarly-reflection-and-concentration-capstone-course/	**Concentration**/Pathway/Project/Track	Medical Education Scholarly Reflection and Concentration Capstone Course	All 4 years	M1-M4	Four-year scholarly concentration allowing students to personalize their curriculum in an area of choice, including medical education. Students gain conceptual understanding and practical skills in research methods and execute an individual capstone project.	Partial	✓	-	✓	✓
Geisel School of Medicine at Dartmouth	Private	https://geiselmed.dartmouth.edu/mes/	Other – Scholars Program	Medical Education Scholars	All 4 years	M1-M4	Longitudinal, student-run scholars program with entry points beginning in M1. Participants attend medical education grand rounds, organize study sessions and reviews for M1 and M2 students, attend a didactic seminar series, and produce a scholarly project by the end of M4.	Full	✓	✓	✓	✓
Georgetown University School of Medicine	Private	https://som.georgetown.edu/medicaleducation/longitudinal-academic-track/merst/	Concentration/Pathway/Project/**Track**	Medical Education Research Scholar Track	All 4 years	M1-M4	Longitudinal program ‘combining education research grand rounds, self-study, interactive didactic sessions in a one-week intensive seminar, and a medical education scholarly project.’ Students also mentor other students new to the program.	Partial	✓	-	✓	✓
Harvard Medical School	Private	https://medcatalog.harvard.edu/courselist.aspx?dep=260	Elective	Medical Education Longitudinal Elective	>4 weeks – 1 year	M3 or M4	Eight-month elective including direct teaching (≥20 hours), a medical education seminar series, observation sessions, and the generation of a ‘final experiential project.’	Full	✓	✓	✓	✓
Icahn School of Medicine at Mount Sinai	Private	https://icahn.mssm.edu/files/ISMMS/Assets/Research/IME/2021-DIME-Application-Guide.pdf	Distinction in Medical Education	Distinction in Medical Education	Variable	Variable	Recognition for ‘graduating students who have taken leadership roles in medical education and have developed and implemented a scholarly project in medical education’ over the course of their undergraduate medical education. A scholarly project is required; direct teaching is one domain that can satisfy the distinction but is not required.	Partial	✓	✓	✓	-
Indiana University School of Medicine	Public	https://medicine.iu.edu/md/curriculum/scholarly-concentrations/medical-education	**Concentration/**Pathway/Project/Track	Medical Education Scholarly Concentration	All 4 years	M1-M4	Four-year scholarly concentration with medical education didactic sessions and a required medical education scholarly project. Students also participate in ‘microteaching’ through a pedagogical methods course.	Full	✓	✓	✓	✓
John A. Burns School of Medicine at the University of Hawaii at Mānoa	Public	https://jabsom.hawaii.edu/wp-content/uploads/2014/09/SeniorHandbook2010-2011.pdf	Elective	Medical Education	>4 weeks – 1 year	M4	Eight- to fourteen-week M4 elective where students serve as PBL tutors. Elective includes a training workshop on selected issues in medical education that includes practice and feedback.	Partial	✓	✓	-	✓
Johns Hopkins University School of Medicine	Private	https://www.hopkinsmedicine.org/som/curriculum/genes_to_society/year-one/scholarly-concentrations.html	**Concentration**/Pathway/Project /Track	Humanism, Ethics, Education, and the Arts of Medicine Scholarly Concentration	>1 year (but not all 4)	M1-M2	Scholarly concentration offering that spans M1-M2 and includes a faculty-mentored scholarly experience culminating in a final presentation.	Partial	✓	-	✓	-
		https://www.hopkinsmedicine.org/som/education-programs/md-program/curriculum-and-degrees/combined-degree-programs.html	Dual Degree – MD/MEHP (Master of Education in the Health Professions)									
Keck School of Medicine of the University of Southern California	Private	https://keck.usc.edu/education/md-program/curriculum/ https://keck.usc.edu/research/research-training-education/	Concentration/Pathway/**Project/**Track	Required Scholarly Project	All 4 years	M1-M4	Required scholarly project as a ‘longitudinal experience throughout all four years of medical school’; in the post-clerkship phase, an area of emphasis in medical education is offered. Students produce a hypothesis-driven scholarly work.	Partial	✓	-	✓	-
Lewis Katz School of Medicine at Temple University	Private	https://medicine.temple.edu/education/md-program/curriculum/year-4	Concentration/Pathway/**Project/**Track	Scholarly Project	>4 weeks – 1 year	M4	Optional, yearlong, mentored scholarly project during M4 culminating in a poster presentation. Students have the option of performing research in an educational domain.	Partial	✓	-	✓	-
Loyola University Chicago Stritch School of Medicine	Private	https://ssom.luc.edu/regrec/elective-catalog/course-catalog/medicaleducation/mded-250/	Elective	Peer Teaching & Mentoring in Medical Education	>4 weeks – 1 year	M2 or M3 or M4	Yearlong M2, M3, or M4 elective beginning with ‘guided didactic/simulation activities’ followed by ‘five small group facilitations with under-classmates.’ Students also complete a ‘learning/teaching self-assessment’ and attend a debrief session.	Partial	✓	✓	-	✓
McGovern Medical School at The University of Texas Health Science Center at Houston	Public	https://med.uth.edu/oep/wp-content/uploads/sites/70/2019/04/Rev_Medical-Education-SC-Curriculum-Requirements-2019-1.pdf	**Concentration/**Pathway/Project/Track	Medical Education Scholarly Concentration	All 4 years	M1-M4	Longitudinal program spanning M1-M4; students attend medical education journal club and workshops, participate in direct teaching, and produce a mentored scholarly project. Optionally, students can also participate in curriculum committee meetings.	Full	✓	✓	✓	✓
Medical College of Georgia at Augusta University	Public	https://augustauniversity.app.box.com/s/xyp1k4l6dp0w5vjc55xnbmu0qfz5o42l	Elective	Medical Education	4 weeks	M3 or M4	Four-week M3 or M4 elective providing a basic foundation in the principles of medical education, with medical education curriculum, teaching, and scholarship serving as the three core components of the course.	Full	✓	✓	✓	✓
Medical College of Wisconsin	Private	https://www.mcw.edu/education/medical-school/discovery-curriculum/Office-of-Student-Scholarly-Activities/pathways/clinician-educator-pathway	Concentration/**Pathway/**Project/Track	Clinician Educator Scholarly Pathway	>1 year (but not all 4)	M1-M3	Scholarly pathway required of all students and integrated within M1-M2, with the option to continue into M3; ‘clinician educator’ available among the pathways to choose from. Students participate in peer tutoring and curriculum development, learn principles of adult learning, and produce a scholarly educational project.	Full	✓	✓	✓	✓
Medical University of South Carolina College of Medicine	Public	https://medicine.musc.edu/education/medical-students/student-affairs/msrp/flex-phase/physician-as-teacher-track	Concentration/Pathway/Project/**Track**	Physician as Teacher Track	>4 weeks – 1 year	M2	Three-month, required scholarly concentration during M2 with ‘Physician as Teacher’ track available. Students produce and present a scholarly project and are exposed to a number of principles of medical educational theory through the track objectives.	Partial	✓	-	✓	✓
New York Medical College	Private	https://www.nymc.edu/school-of-medicine-som/som-academics/undergraduate-medical-education-md-program/curriculum/areas-of-concentration-program-/	**Concentration**/Pathway/Project/Track	Medical Education Area of Concentration	All 4 years	M1-M4	Optional area of concentration spanning from pre-clerkship through M4. Foundational elective course, seminars, advanced elective, and scholarly capstone project offered for the medical education discipline. Students also have the opportunity to refine their teaching skills across varying teaching venues at the medical school.	Full	✓	✓	✓	✓
New York University Grossman School of Medicine	Private	https://med.nyu.edu/education/md-degree/md-curriculum/stage-three-individualized-exploration/scholarly-concentrations	**Concentration/**Pathway/Project/Track	Medical Education Scholarly Concentration	>4 weeks – 1 year	M4	Standard scholarly concentration lasting 12 weeks in M4, with a concentration area in medical education offered. Students prepare a mentored scholarly project and are encouraged to submit their work to a professional journal. Students have the option of spending 4 of the 12 weeks in a selective or elective course; an available Practice of Medicine Teaching Academy elective provides the opportunity for direct teaching through twelve teaching session blocks and includes required ‘instructor development activities.’ Together, these activities constitute a full CETL.	Full	✓	✓	✓	✓
Oakland University William Beaumont School of Medicine	Public	https://www.oakland.edu/Assets/Oakland/medicine/files-and-documents/students/course%20description%20catalog%2020182020.v2.pdf	Elective	Medical Education	4 weeks	M4	Four-week elective introducing students to ‘contemporary thinking on medical education and leadership’ through reading, seminars, online discussions, and a final presentation and essay. Course also includes a ‘microteaching experience’ where students teach peers and instructors about a topic of medical education of their choice.	Partial	✓	✓	-	✓
Oregon Health & Science University School of Medicine	Public	https://www.ohsu.edu/sites/default/files/2020-08/Course%20Catalog%20AY2021%20FINAL%208.19.20.pdf	Elective	Medical Education with the Wy’East Post-Bac Program	4 weeks	M3 or M4	Four-week elective in which M3 and M4 students work with students from Native American backgrounds in the Wy’East post-baccalaureate program. Students design and lead small group sessions, receive close faculty mentorship, and complete ‘new faculty modules created by the Teaching and Learning Center at OHSU.’	Partial	✓	✓	-	✓
Penn State College of Medicine	Public	https://med.psu.edu/md/accelerated/clinician-educator	Dual Degree (MD/MEd – Medical and Health Professions Education)									
Perelman School of Medicine at the University of Pennsylvania	Private	https://www.med.upenn.edu/student/certificate-programs.html	**Concentration/**Pathway/Project/Track	Medical Education Area of Concentration	Unspecified	‘Upper-level students’	Longitudinal program for upper-level students including a ‘Frontiers in Medical Education’ didactic series, journal club, direct teaching and medical education leadership, a curriculum development or medical education research project, and a final capstone presentation.	Full	✓	✓	✓	✓
Renaissance School of Medicine at Stony Brook University	Public	https://renaissance.stonybrookmedicine.edu/ugme/education/Scholarly	**Concentration**/Pathway/Project/Track	Scholarly Concentrations Program – Medical Education Track	All 4 years	M1-M4	Four-year scholarly concentration offering with medical education as an established track. Students participate in curriculum development and evaluation, complete various teaching activities (20 hours), produce a scholarly project, and attend medical education seminars.	Full	✓	✓	✓	✓
Rutgers New Jersey Medical School	Public	https://njms.rutgers.edu/admissions/distinction_program.php	Distinction in Medical Education	Distinction Program in Medical Education	All 4 years	M1-M4	Longitudinal program exposing students over four years to the ‘governance, structure and functions of academic medicine.’ Incorporates didactic sessions and direct teaching and mentoring; students also produce a scholarly capstone project.	Full	✓	✓	✓	✓
Rutgers, Robert Wood Johnson Medical School	Public	https://rwjms.rutgers.edu/education/medical_education/distinction/medical_edu.html	Distinction in Medical Education	Distinction in Medical Education Program	All 4 years	M1-M4	Four-year program incorporating medical education electives, direct teaching, a scholarly project, and an accompanying conference submission or presentation.	Full	✓	✓	✓	✓
Sidney Kimmel Medical College at Thomas Jefferson University	Private	https://www.jefferson.edu/academics/colleges-schools-institutes/skmc/undergraduate-medical-education/curriculum/Scholarly-Inquiry/Scholarly-Inquiry-Tracks.html	Concentration/Pathway/Project/**Track**	Medical Education Scholarly Inquiry Track	All 4 years	M1-M4	All students select a ‘scholarly inquiry track’ spanning M1-M4; medical education is among the tracks available. Students are exposed to principles of adult learning, produce medical education scholarship, and engage in experiential learning to apply their skills in teaching.	Full	✓	✓	✓	✓
Stanford University School of Medicine	Private	http://med.stanford.edu/meded.html	**Concentration/**Pathway/Project/Track	Scholarly Concentration: Medical Education	All 4 years	M1-M4	Option within scholarly concentration program required of all students that runs longitudinally. Students complete 12 total coursework units related to medical education, including options in the Graduate School of Education, produce a scholarly project, and are encouraged to serve as a teaching assistant.	Full	✓	✓	✓	✓
State University of New York Downstate Health Sciences University College of Medicine	Public	https://sls.downstate.edu/admissions/com/curriculum/med-educator-path.html	Concentration/**Pathway/**Project/Track	Medical Educator Pathway	All 4 years	M1-M4	Longitudinal pathway in which students serve as ‘near peer educators,’ develop educational materials and test items, work on scholarly projects, and attend medical education classes and faculty development workshops.	Full	✓	✓	✓	✓
State University of New York Upstate Medical University College of Medicine	Public	https://www.upstate.edu/currentstudents/document/course_selection_com.pdf	Elective	Rural Medicine Curriculum Design Elective	>1 year (but not all 4)	M1-M2 plus M4	Elective course spanning M1 and M2 and resuming in M4. Students work to develop curricula and ‘play a lead role in program evaluation and development’ for an array of rural health courses at the medical school. Students also lead discussions in the electives and perform educational research including manuscript preparation and presentation at conferences.	Partial	✓	✓	✓	-
Texas A&M University College of Medicine	Public	https://medicine.tamu.edu/omsre/msrpp.html	Concentration/**Pathway**/Project/Track	Medical Scholar Research Pathway Program	Variable	Variable	Voluntary research program with medical education offered as one of nine ‘areas of research.’ Students can choose from three ‘pathways’, which vary in length from 4–8 week blocks to a full non-credit gap year between M3 and M4. Regardless of pathway, students in the program produce a scholarly research product under faculty supervision.	Partial	✓	-	✓	-
		https://medicine.tamu.edu/degrees/documents/md-ms-edhp.pdf	Dual Degree – MD/MS Education for Healthcare Professionals (EDHP)									
The George Washington University School of Medicine and Health Sciences	Private	https://smhs.gwu.edu/oso/track-program/medical-education-leadership	**Concentration**/Pathway/Project/Track	Medical Education and Leadership Scholarly Concentration	All 4 years	M1-M4	Longitudinal program including small group teaching, an ‘experiential opportunity’ during M1 summer, a scholarly project in M3-M4, and a variety of workshop series and educational electives. A number of medical education leadership opportunities are also available.	Full	✓	✓	✓	✓
The Robert Larner, M.D. College of Medicine at the University of Vermont	Public	https://www.med.uvm.edu/mededucation/teachingscholarly	Required	Teaching Practicum	4 weeks	M4	Required practicum that can be fulfilled either by completing a teaching practicum or a scholarly project. In the teaching practicum, students serve as a teaching assistant, attend teaching workshops, and develop educational materials.	Partial	✓	✓	-	✓
The University of Arizona College of Medicine – Tucson	Public	https://medicine.arizona.edu/education/md-program/distinction-tracks/med-ed-track	Concentration/Pathway/Project/**Track**	Medical Education Distinction Track	All 4 years	M1-M4	Longitudinal track involving students in teaching, curriculum development, assessment, mentoring, educational leadership and administration, and required and elective coursework on educational theory. Students produce and present a scholarly capstone project.	Full	✓	✓	✓	✓
The University of Chicago Pritzker School of Medicine	Private	https://pritzker.uchicago.edu/academics/medical-education-track	Concentration/Pathway/Project/**Track**	Medical Education Scholarship Track	>1 year (but not all 4)	M1-M2 plus M4	Longitudinal scholarship track exposing students to principles of health professions education through ‘active experiences, reflection, and group learning.’ Students can participate in various teaching opportunities and produce a scholarly research project.	Full	✓	✓	✓	✓
The University of Texas at Austin Dell Medical School	Public	https://catalog.utexas.edu/medical/degrees/dualdegrees/	Dual Degree – MD/MEd									
The University of Texas Health Science Center at San Antonio Joe R. and Teresa Lozano Long School of Medicine	Public	https://www.uthscsa.edu/academics/medicine/education/ume/distinction-medical-education	Distinction in Medical Education	Distinction in Medical Education	Variable	Variable	Distinction program requiring, during the course of medical school, academic medicine professional development activities and a scholarly project and poster presentation totaling at least 500 hours of ‘scholarly activity.’	Partial	✓	-	✓	-
The University of Texas Southwestern Medical School	Public	https://d2l.utsouthwestern.edu/d2l/lor/viewer/viewFile.d2lfile/6606/3766,-1/	Elective	Medical Education Fourth-Year Elective	>4 weeks – 1 year	M4	Seven-month M4 elective with regular interactive teaching sessions, hands-on teaching activities, journal club, and a scholarly final project. Culminates with students assembling a personal teaching portfolio.	Full	✓	✓	✓	✓
The University of Toledo College of Medicine and Life Sciences	Public	https://www.utoledo.edu/med/md/curriculum/curriculum4/four_week_electives.html	Elective	Teaching Elective	4 weeks	M4	Elective for M4 students to provide clinical instruction to M1, M2, and/or M3 students. Students also shape instruction at the medical school through innovation in developing a new ‘learning module’ aligned with institutional and NBME standards.	Full	✓	✓	✓	✓
The Warren Alpert Medical School of Brown University	Private	https://education.med.brown.edu/scholarly-concentrations-program/medical-education	**Concentration**/Pathway/Project/Track	Scholarly Concentration in Medical Education	All 4 years	M1-M4	Longitudinal program involving students in curriculum development, regular teaching and learning seminars, and direct teaching. Students may also complete an independent study to prepare a scholarly final project.	Full	✓	✓	✓	✓
Tufts University School of Medicine	Private	https://medicine.tufts.edu/education/doctor-medicine/curriculum-overview	Required	Student-As-Teacher	All 4 years	M1-M4	Longitudinal program ‘expos[ing] all students to basic principles of teaching and learning at different points in their four-year medical school training’ through online learning modules and a direct ‘field teaching experience.’	Partial	✓	✓	-	✓
Universidad Central del Caribe School of Medicine	Private	https://www.uccaribe.edu/wp-content/uploads/2021/10/UCC-SENIOR-ELECTIVE-CATALAGO-2021-22-v20210430.pdf	Elective	Deanship in Medicine: Student as Teacher	4 weeks	M4	Two- or four-week M4 elective in which students participate in didactic sessions on learning theory, teaching techniques, and methods of delivering feedback. Students also serve as instructors for and evaluate junior medical students in clinical skills courses.	Partial	✓	✓	-	✓
University of California, Riverside School of Medicine	Public	https://ume.ucr.edu/sites/g/files/rcwecm4856/files/2021-03/som_course_catalog_21.pdf	Elective	Experience in Undergraduate Medical Education	4 weeks	M4	M4 elective lasting up to four weeks in which students facilitate classes for junior medical students, prepare educational materials, and review medical education research.	Partial	✓	✓	-	✓
University of California, San Francisco School of Medicine	Public	https://meded.ucsf.edu/faculty-educators/faculty-development/advanced-education-programs/pathway-discovery-health-professions-education	Concentration/**Pathway/**Project/Track	Pathway to Discovery in Health Professions Education	>1 year (but not all 4)	M3-M4	Pathway program open to both senior medical students and residents and fellows in which participants obtain hands-on teaching experience, participate in courses, and complete a scholarly project.	Full	✓	✓	✓	✓
University of Cincinnati College of Medicine	Public	https://med.uc.edu/education/medical-student-education/academic-support/programs/tutoring	Other – Multi-Year Course	Teaching in Medical Education	All 4 years	M1-M4	Longitudinal course spanning M1-M4 in which students are exposed to learning theory and engage in at least 80 hours of teaching activity.	Partial	✓	✓	-	✓
University of Colorado School of Medicine	Public	https://ucdenver.oasisscheduling.com/public/courses/index.html?yid=2022&slid=2	Elective	Physician as Educator	4 weeks	M4	Four-week M4 elective to develop students’ teaching skills in both clinical and classroom settings. Students participate in teaching workshops, complete an OSTE, and complete a variety of teaching experiences.	Partial	✓	✓	-	✓
University of Florida College of Medicine	Public	https://discovery.education.med.ufl.edu/discovery-tracks/medical-education/	Concentration/Pathway/Project/**Track**	Medical Education Track	All 4 years	M1-M4	Mentored research program in medical education spanning M1-M4. Students develop a robust ‘individual planned program’ and produce and present a piece of educational scholarship. Direct teaching opportunities are also available.	Full	✓	✓	✓	✓
University of Illinois College of Medicine	Public	https://chicago.medicine.uic.edu/education/md-curriculum/curriculum-by-year/phase-2-3/electives-and-pathway-structure/electives-catalog/m4-teaching-hospital-practicum-ui-health/	Elective	M4 Teaching – Immersion	2 weeks or less	M4	Elective for M4 students to teach, observe, and provide feedback to junior medical students in clinical settings. Participants attend mandatory didactic sessions on teaching at the bedside and providing feedback to learners.	Partial	-	✓	-	✓
University of Iowa Roy J. and Lucille A. Carver College of Medicine	Public	https://medicine.uiowa.edu/md/curriculum/distinction-tracks/teaching-distinction-track	Concentration/Pathway/Project/**Track**	Teaching Distinction Track	>1 year (but not all 4)	M2-M4	Medical education track requiring students to complete at least 60 hours of direct teaching, develop a ‘substantive curriculum project that can be used for future as well as current teaching activities,’ and assemble an educational portfolio. Students also sit on educational committees and complete a ‘Teaching Skills for Medical Students’ elective.	Full	✓	✓	✓	✓
University of Louisville School of Medicine	Public	https://louisville.edu/medicine/distinction/tracks/dime	Distinction in Medical Education	Distinction in Medical Education	All 4 years	M1-M4	Spanning M1-M4, this program involves students in direct teaching, instructional content development, and a medical education scholarly research project. Students also ‘master the content and principles of medical teaching and learning through assigned readings.’	Full	✓	✓	✓	✓
University of Michigan Medical School	Public	https://medicine.umich.edu/medschool/education/md-program/curriculum/impact-curriculum/paths-excellence/scholarship-learning-teaching	Concentration/**Pathway/**Project/Track	Scholarship of Learning & Teaching Path of Excellence	All 4 years	M1-M4	Longitudinal program exposing students to key principles of health professions education. Students choose a ‘focus domain’ in either learning, teaching, or medical education scholarship. The final ‘Capstone for Impact’ project is of suitable quality for presentation at a professional conference or publication in a peer-reviewed journal.	Full	✓	✓	✓	✓
University of Minnesota Medical School	Public	https://med.umn.edu/md-students/individualized-pathways	Research Program	Summer Internship in Medical Education	>4 weeks – 1 year	M1 Summer	Eight-week summer internship offered to students finishing M1. Students are exposed to medical education theory through readings, journal club, and one-on-one meetings with faculty. Students also produce a scholarly project that ‘reflect[s] a priority issue within the medical school’s Office of Medical Education.’	Partial	✓	-	✓	✓
University of Nebraska College of Medicine	Public	https://www.unmc.edu/com/education/md-enrichment/emet.html	Concentration/Pathway/Project/**Track**	Clinical Educator Enhanced Medical Education Track	All 4 years	M1-M4	Four-year curriculum exposing students to principles of adult learning and engaging them in teaching and evaluation exercises. Students ‘create a capstone research project or curriculum element and convert it into scholarship.’	Full	✓	✓	✓	✓
University of Nevada, Reno School of Medicine	Public	https://med.unr.edu/office-of-medical-research/scholarly-concentration/scholarly-concentration-in-medical-education	**Concentration/**Pathway/Project/Track	Scholarly Concentration in Medical Education	All 4 years	M1-M4	Intensive, longitudinal program engaging students over four years as Reporter (learn about), Interpreter (do), Manager (refine), and Educator (demonstrate scholarship) within medical education. Includes lectures, webinars, or conferences related to medical education, direct teaching, and the generation and presentation of a scholarly project.	Full	✓	✓	✓	✓
University of New Mexico School of Medicine	Public	https://hsc.unm.edu/medicine/education/md/_docs/omsa/current-students/class-of-2021/2020-2021-phase-iii-clerkship-catalog—covid-edition.pdf	Elective	WISE/Doctoring Teaching Elective	4 weeks	M4	Four-week M4 elective in which students perform hands-on teaching of junior medical students and develop curricula and educational materials by applying principles of educational theory (e.g., Kern’s 6-Step Approach).	Full	✓	✓	✓	✓
University of North Carolina School of Medicine	Public	https://www.med.unc.edu/md/curriculum/primary-care-programs-and-scholarly-tracks/scholarly-concentration-program/medical-education-scholarly-concentration/	**Concentration/**Pathway/Project/Track	Medical Education Scholarly Concentration	All 4 years	M1-M4	Longitudinal scholarly concentration in medical education in which students attend regular medical education events and seminars, participate in direct teaching or an educational leadership opportunity, and complete an independent study or research project.	Full	✓	✓	✓	✓
University of North Dakota School of Medicine and Health Sciences	Public	https://med.und.edu/education-resources/electives/_files/docs/med-9508.pdf	Elective	Medical Education	4 weeks	M4	Elective for M4 students to serve as ‘novice teachers’ facilitating small group learning and presenting a lecture. Students are exposed to principles of adult learning theory. Students also produce test items, attend journal club, ‘intern’ on the academic faculty staff, and produce an educational improvement ‘mini-project.’	Partial	✓	✓	-	✓
University of Puerto Rico School of Medicine	Public	https://md.rcm.upr.edu/curriculum/download/fourth-year-manual-and-elective-course-catalog/	Elective	Peer to Peer in Medical Education	2 weeks or less	M4	M4 elective ‘designed to develop knowledge and skills in medical education … through lectures, workshops, and supervised practice, students will develop small group teaching skills, seminar facilitation, and [prepare] didactic materials.’	Partial	-	✓	-	✓
University of Rochester School of Medicine and Dentistry	Private	https://www.urmc.rochester.edu/education/md/admissions/why-rochester/elective-pathways.aspx#MedicalEducationPathway	Concentration/**Pathway/**Project/Track	Medical Education Pathway	>1 year (but not all 4)	M2-M4	Pathway program spanning at least two years in which students prepare and deliver a lecture to a small group, serve as a PBL tutor, write learning objectives and test items, and participate in faculty development workshops, journal clubs, and educational theory seminars.	Partial	✓	✓	-	✓
University of South Carolina School of Medicine, Columbia	Public	https://uscmed.oasisscheduling.com/public/courses/index.html?yid=2022&slid=1	Elective	Medical Education: Learning From Teaching	>4 weeks – 1 year	M4	Semester-long elective where M4 students serve as instructors for educational activities in an Introduction to Clinical Medicine course. Also includes an orientation and didactic sessions related to the teaching responsibilities.	Partial	✓	✓	-	✓
University of South Dakota Sanford School of Medicine	Public	https://www.usd.edu/medicine/scholarship-pathways	Concentration/**Pathway/**Project/Track	Medical Education Scholarship Pathway	All 4 years	M1-M4	Elective, four-year ‘scholarship pathway’ with medical education offered as one of three categories; the final product is a manuscript suitable for publication. ‘Students further explore their selected topic through their choice of electives, self-directed learning, faculty mentorship, periodic seminars and independent work.’	Partial	✓	-	✓	✓
University of Utah School of Medicine	Public	https://medicine.utah.edu/students/programs/md/curriculum/students-as-teachers.php	Concentration/**Pathway/**Project/Track	Students as Teachers Pathway	All 4 years	M1-M4	Four-year elective pathway exposing students to adult learning theory, small group teaching, curriculum, providing feedback to learners, and career development as a clinician educator. Students also produce a scholarly capstone project.	Full	✓	✓	✓	✓
USF Health Morsani College of Medicine	Public	https://health.usf.edu/medicine/mdprogram/scp/eSC	**Concentration**/Pathway/Project/Track	Medical Education Scholarly Concentration	All 4 years	M1-M4	Elective scholarly projects program with a Medical Education offering. Students attend regular meetings in M1 and M2, produce an ‘educational reflection paper’ in M3, and finish by completing 90 hours of direct teaching, attending didactic sessions on effective teaching, and producing a final scholarly project.	Full	✓	✓	✓	✓
Vanderbilt University School of Medicine	Private	https://peabody.vanderbilt.edu/degrees-programs/mdmed_joint_degree_program.php	Dual Degree – MD/MEd									
		https://medschool.vanderbilt.edu/md-admissions/students-as-teachers-cohort/	Elective	Students as Teachers	>4 weeks – 1 year	M3-M4	Yearlong elective including workshops on educational theory – some of which are student-led – and direct teaching (40 hours) in anatomy lab and group reviews.	Partial	✓	✓	-	✓
Virginia Tech Carilion School of Medicine	Public	https://medicine.vtc.vt.edu/content/dam/medicine_vtc_vt_edu/student-life/catalog/2020-2021-course-catalog.pdf	Elective	Medical Education	2 weeks or less	M4	Students in this two-week M4 elective learn to facilitate small group learning, write cases for education and evaluation, and participate in basic curriculum design and development.	Partial	-	-	-	✓
Wake Forest School of Medicine of Wake Forest Baptist Medical Center	Private	https://school.wakehealth.edu/Education-and-Training/Certificate-Programs/Medical-Education-Certificate-Program	Other – Certificate Program	Medical Education Certificate Program	>4 weeks – 1 year	M2 or M3 or M4	Optional certificate program spanning the academic year of M2, M3, or M4; also open to PA and CRNA students. Provides ‘training in adult learning principles and evidence-based instructional design and delivery’ relevant to medical education.	Partial	✓	-	-	✓
Washington State University Elson S. Floyd College of Medicine	Public	https://app.medicine.wsu.edu/pdf/Scholarly-Projects-Student-Manual_6_9_2020.pdf	Concentration/Pathway/**Project/**Track	Medical Education and Community Education Scholarly Project	>1 year (but not all 4)	M2-M4	Required scholarly project spanning M2-M4 with an established ‘Medical Education and Community Education’ category offered. Students produce a mentored, question-driven scholarly work as part of an academic portfolio submitted in M4.	Partial	✓	-	✓	-
Weill Cornell Medicine	Private	https://medicaleducation.weill.cornell.edu/medical-education/md-program/areas-concentration	**Concentration/**Pathway/Project/Track	Medical Education Area of Concentration	All 4 years	M1-M4	Required Area of Concentration Program with an offering in medical education available. Student exploration begins in M1; scholarly project preparation commences in M3 and continues into M4. Students ‘obtain in-depth knowledge’ and complete a series of ‘core activities’ specific to the discipline of their AOC.	Partial	✓	-	✓	✓
Western Michigan University Homer Stryker M.D. School of Medicine	Private	https://med.wmich.edu/sites/default/files/2019%20Curriculum%20Brochure%20FINAL.pdf	Elective	Selected Topics in Medical Education Early Electives	4 weeks	M1-M2	Set of four one-week electives offered during M1 and M2 ‘early elective’ blocks that collectively provide exposure to curriculum development, direct teaching, a research project ‘on the impact of teaching strategies on medical student learning,’ and structured discussion on ‘scholarly literature on learning and instructional design.’	Full	✓	✓	✓	✓
Wright State University Boonshoft School of Medicine	Public	http://f5webserv.wright.edu/bsom/course_catalog/All_BSOM_Courses.pdf	Elective	Academic Medicine – General Medical Education	4 weeks	M3 or M4	Four-week, customizable medical education M3/M4 elective that allows students to participate in small-group direct teaching, conduct medical education research, and learn principles of adult learning theory.	Full	✓	✓	✓	✓
Yale School of Medicine	Private	https://medicine.yale.edu/education/curriculum/advancedtraining/clinicalelectives/visitingelectives/teachingandlearningcenter/	Elective	Teaching and Learning Center Medical Education Elective	2 weeks or less	M3 or M4	Two-week M3 or M4 elective incorporating ‘didactic lectures, observations, group exercises, and teaching activities.’ Culminates with the completion of a set of OSTEs.	Partial	-	✓	-	✓

Note *to editors: this table may be found in the separately submitted Excel file*

### CETL program components

**Figure 3.**
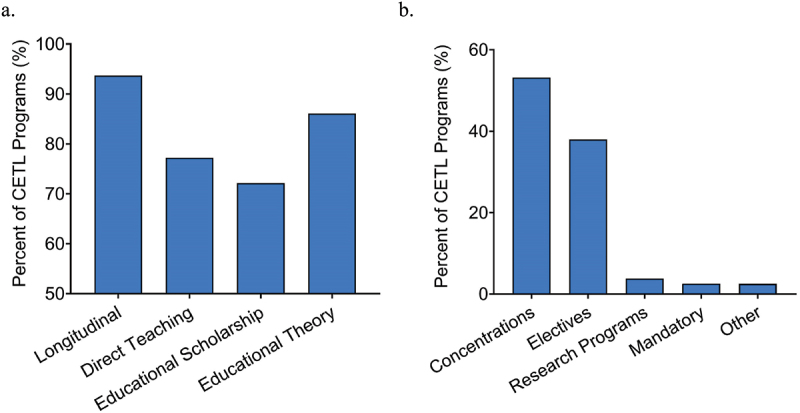
(a) Percent of clinician-educator track-like (CETL) programs (n = 79) that fulfill each of the CETL criteria. (b) CETL program classification (n = 79). ‘Concentrations’ encompasses formal certificates, concentrations, distinctions, pathways, projects, and tracks.

We aimed to understand how CETLs differ according to their program components (longitudinal, direct teaching, educational scholarship, educational theory). Of the 79 CETLs, the 39 full CETLs contain all 4 components. The 40 partial CETLs include 26 that have 3 CETL components. Seventeen longitudinal programs incorporate direct teaching and educational theory, 7 longitudinal programs incorporate educational scholarship and educational theory, and 2 longitudinal programs incorporate direct teaching and educational scholarship. Twelve CETLs have 2 components. Eight longitudinal programs incorporate educational scholarship, 3 non-longitudinal programs incorporate direct teaching and educational theory, and 1 longitudinal program incorporates educational theory. Two partial CETLs contain only 1 component (1 educational scholarship program and 1 educational theory program). Overall, 94% (74/79) of CETLs are longitudinal, and educational theory (86%, 68/79) is more prevalent than direct teaching (77%, 61/79) and educational scholarship (72%, 57/79) (Figure 3A).

### CETL classification, length, and timing

We classified CETLs according to shared characteristics and naming conventions (Figure 3B). Most often (52%, 41/79), CETLs are designated as formal concentrations or similar structures: concentrations (20%, 16/79), pathways (10%, 8/79), tracks (10%, 8/79), distinctions (6%, 5/79), projects (5%, 4/79), or certificates (1%, 1/79). Collectively, these CETLs are academic experiences promoting in-depth learning in a topical area of focus and are usually but not always distinct from core medical school curricula.

Thirty of the 79 (38%) CETLs are designated as electives, the majority of which occur exclusively during the fourth year of medical school (70%, 21/30). Three of the 79 (4%) CETLs are research programs. Two of these occur during the summer between the first and second year of medical school. One occurs during the third year. Two of the 79 (3%) CETLs are school requirements (a 4-year Student-As-Teacher program and a month-long Teaching Practicum). The remaining 2 (3%) CETLs represent uniquely branded experiences: a Scholars program and a 4-year course not designated as an elective.

**Figure 4.**
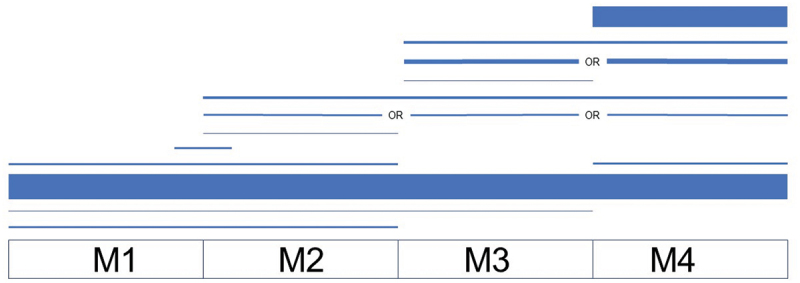
Clinician-educator track-like (CETL) program timing chart (n = 75). Line thickness represents the number of schools. Four schools are excluded from the visualization as their programs may occur at an unspecified or variable time during M1 – M4 (3) or unspecified time during ‘upper years’ (1).

CETLs vary tremendously in length. A plurality of programs (37%, 29/79) span all 4 years of medical school. Eighteen of the 79 CETLs (23%) last 4 weeks and 15 (19%) are between 4 weeks and 1 year. A smaller number of programs are longer than 1 year but shorter than 4 years (10%, 8/79) and even fewer are shorter than 4 weeks (6%, 5/79). Timing of CETLs during medical school is also quite heterogeneous. Although a majority of CETLs start in the first year and continue through the fourth year (37%, 29/79) or occur exclusively during the fourth year (30%, 24/79), programs occur at all points during medical school (Figure 4).

### CETLs by institution characteristics

We additionally analyzed whether CETL program availability is associated with institutional geographic location, public or private status, and ranking among medical schools according to U.S. News & World Report [42].

Medical schools were geographically divided into Midwest (23%, 35/155), Northeast (23%, 36/155), South (37%, 57/155), West (15%, 23/155), or Territorial (3%, 4/155), according to U.S. Census Bureau definitions [43]. A higher proportion of Northeast institutions have CETLs (69%, 25/36) compared to institutions in the South (37%, 21/57, p < 0.05). Other geographic regions do not significantly differ from each other in CETL availability. Public (45/93, 48%) and private (34/62, 55%) institutions offer CETLs at similar rates. With respect to third-party ranking, the top 50 medical schools for research according to U.S. News & World Report more commonly have CETL programs (65%, 33/51) than unranked schools and schools ranked 51 or above (44%, 46/104, p < 0.05).

### Dual degree programs

Among the 116 schools without a full CETL, 4% (n = 5) offer a dual degree program for medical students. The dual degree programs encompass 2 general education programs and 3 health professions education programs.

## Discussion

Our web search provides an exploratory look at the current state of CETLs in UME. The search algorithm was reproducible, with a median percent agreement between raters of 87% (range across rater pairs: 80%-100%), despite 3–6 months separating the 2 independent searches and program availability changing throughout the time period. Approximately half (51%, 79/155) of U.S. allopathic medical schools have identifiable structures to prepare their students for careers as clinician-educators. Prior works demonstrate that formal training in educational theory, curriculum design, scholarship, and teaching produces better educators [44–46] as well as better learners [47]. Medical students soon will be residents and faculty whose educator competencies will benefit trainees and patients alike [47]. These constitute compelling reasons for all U.S. allopathic medical schools to implement – and continually improve – CETLs and dual degree programs in education for their medical students.

Most commonly, CETLs address the clinician-educator competency of educational theory, are formally organized as concentrations (or analogous structures), and span all four years of medical school. Nevertheless, programs exhibit significant heterogeneity in their naming, timing, and content. Friedman and colleagues [16] report on heterogeneity in the timing of CETs in graduate medical education (GME), proposing that future research examine the type and amount of time required in CETs for sufficient clinician-educator skill-building and professional identity formation. We encourage similar research for CETLs in UME. Applying the community of practice framework [24,25], a greater number of hours devoted to a CETL or dual degree program in education may result in deeper interactions within the medical education community of practice, stronger professional identity formation, and a higher likelihood for medical students to become clinician-educators. Clinician-educators commonly describe their entry into the position as serendipitous and characterize the career pathway as ill-defined [48]. This underscores the critical role that time-intensive CETLs could have in spurring early entry into the clinician-educator career pathway. Future research should explore this role by assessing the long-term impact of CETLs and dual degree programs in education on career trajectory.

With CETLs and CETs increasing in UME and GME, respectively, coordination is necessary to promote continuous development along the educator skill spectrum and reduce curricular redundancy across these two stages of training. Soriano and colleagues [32] found that UME student-as-teacher programs have remarkably similar content and teaching formats to resident-as-teacher programs [49]. A clear framework of educator competencies for both UME and GME may facilitate the alignment of CETLs with CETs to achieve optimal outcomes. The key roles, domains of competence, and core competencies for clinician-educators defined by Sherbino and colleagues [6] may serve as a foundation for institutions seeking to design their own competency frameworks. Standardizing such frameworks would create a shared language for clinician-educator competency building and transform how future clinician-educators are developed. It may therefore behoove educational leadership at institutions with CETs and CETLs to collaborate on a consensus competency framework for use across UME and GME nationwide.

Models of successful CETLs are useful references for institutions aiming to implement their own programs. In recognition of this, our study aims to effectively summarize 79 distinct CETLs representing the diversity of offerings at U.S. allopathic medical schools. Numerous works discuss the challenges of such programs. Faculty doubts about student teaching competence may drive institutional resistance to implementation [32], though students are known to be as good as faculty in certain teaching contexts [50]. It is reasonable to suppose that this capability may extend to other clinician-educator domains. In their report on a scholarly concentrations program at the Vanderbilt University School of Medicine, Gotterer and colleagues [51] describe additional challenges: deciding on program timing, tracking student progress, and funding program expenses, among others. Nevertheless, suggestions for actualizing such programs despite their challenges have also been published. Freret and colleagues [52] describe 12 tips for implementing student-as-teacher programs, including defining program scope, promoting longitudinal trainee and faculty relationships, and collecting feedback from all stakeholders. These tips may be adapted to enhance nascent or existing CETLs. We recommend educational leaders draw from the pool of existing CETLs, anticipate challenges to implementing and maintaining their own program, and adhere to published guidance to maximize the chances of CETL success.

While CETLs are most immediately pertinent to medical students, their relevance may begin before medical school. Celebi and colleagues [53] found that implementation of a CET in their residency program was associated with increased applicant interest, larger application numbers, and enhanced applicant quality. We speculate that CETLs may have a similar impact on medical school admissions, particularly for programs that are prominently featured on medical school institutional websites. The content of residency program websites is known to be important for residency applicants [54], and as most medical school applicants use the Internet as an information source when applying [39], the importance of website advertising likely applies to medical school recruitment as well. In our study, the prevalence of CETLs interestingly varied with institutional geography (more common in the Northern region of the U.S. than the Southeast) and ranking (greater presence at top 50 research institutions than lower-ranked/unranked institutions), but not with institutional status as public or private. Medical school applicants aspiring to become clinician-educators may benefit from awareness of these trends when curating their list of prospective schools.

### Limitations

Several limitations exist for this study. First, our data do not account for CETLs and CETL components that may be in place but are not evident on institutional websites. Despite this, our CETL sample is large: 79 programs representing 51% of all U.S. allopathic medical schools. This exceeds the number of formal medical education training programs reported in similar works [32]. Our data are also descriptive of various CETL characteristics and are as current as institutional websites, although some websites may be outdated. Second, our web search was not sensitive to program information (e.g., outcomes) typically reported by surveys and case reports. For this work, we desired to have a comprehensive and contemporary picture of institutional CETLs at allopathic medical schools across the U.S. Web surveys are known for low response rates [55,56], and CETLs are sparse in the current literature. Nevertheless, further surveying of educational leadership at medical schools and systematic literature review would add invaluable program information and, importantly, clarify the scope of individual CETLs to enable further analysis. Third, website analysis is inherently subjective, and the personalized algorithms of search engines risk the introduction of selection bias [57]. We employed a comprehensive, algorithmic web search strategy to counteract this, with high inter-rater reliability scores indicating search reproducibility and low rater subjectivity. Finally, our definition for CETL was limited and did not include the many ways medical students may prepare for future clinician-educator careers, such as serving on their institution’s curriculum committee and developing courses [11,22]. Future research should explore these opportunities as well as programs that address clinician-educator competencies beyond the scope of our work (e.g., leadership).

## Conclusion

In conclusion, we provide an overview of the current state of clinician-educator track-like programs (CETLs) in undergraduate medical education (UME) as assessed from institutional websites. Only half of U.S. allopathic medical schools appear to have formal structures to prepare their students for careers as clinician-educators. To create a future with a sustainable output of skilled clinician-educators, UME must continue to increase the number and quality of CETLs and dual degree programs in education. Future studies should explore CETL outcomes, including end-of-program learner satisfaction, demonstrated learner proficiency in clinician-educator competencies, and long-term impact on career trajectory.

## Appendix

**Table A1.** Agreement assessment between reviewers performing the web search algorithm.

Reviewer A	Reviewer B	A Yes, B Yes	A Yes, B No	A No, B Yes	A No, B No	% Agreement	Cohen’s Kappa
R.C.B.	M.A.S.	15	5	0	19	87.18%	0.75
M.A.S.	E.S.L.	10	0	4	13	85.19%	0.71
E.S.L.	A.A.A.	6	0	0	8	100.00%	1
R.C.B.	E.S.L.	25	2	1	22	90.38%	0.81
M.A.S.	A.A.A.	8	2	3	12	80.00%	0.59
